# A THP-1 Cell Line-Based Exploration of Immune Responses Toward Heat-Treated BLG

**DOI:** 10.3389/fnut.2020.612397

**Published:** 2021-01-13

**Authors:** Ying Deng, Coen Govers, Ellen ter Beest, Aalt-Jan van Dijk, Kasper Hettinga, Harry J. Wichers

**Affiliations:** ^1^Food and Biobased Research, Wageningen University and Research, Wageningen, Netherlands; ^2^Food Chemistry, Department of Agrotechnology and Food Sciences, Wageningen University and Research, Wageningen, Netherlands; ^3^Bioinformatics Group, Department of Plant Sciences, Wageningen University and Research, Wageningen, Netherlands; ^4^Food Quality and Design, Department of Agrotechnology and Food Sciences, Wageningen University and Research, Wageningen, Netherlands

**Keywords:** β-lactoglobulin, THP-1, macrophage, dendritic cell, heat-treatment, microarray, cytokine, ingenuity pathway analysis

## Abstract

Allergen recognition and processing by antigen presenting cells is essential for the sensitization step of food allergy. Macrophages and dendritic cells are both phagocytic antigen presenting cells and play important roles in innate immune responses and signaling between the innate and adaptive immune system. To obtain a model system with a homogeneous genetic background, we derived macrophages and dendritic cells from THP-1 monocytes. The difference between macrophages and dendritic cells was clearly shown by differences in their transcription response (microarray) and protein expression levels. Their resemblance to primary cells was analyzed by comparison to properties as described in literature. The uptake of β-lactoglobulin after wet-heating (60°C in solution) by THP-1 derived macrophages was earlier reported to be significantly increased. To analyse the subsequent immune response, we incubated THP-1 derived macrophages and dendritic cells with native and differently processed β-lactoglobulin and determined the transcription and cytokine expression levels of the cells. A stronger transcriptional response was found in macrophages than in dendritic cells, while severely structurally modified β-lactoglobulin induced a more limited transcriptional response, especially when compared to native and limitedly modified β-lactoglobulin. These results show that processing is relevant for the transcriptional response toward β-lactoglobulin of innate immune cells.

## Introduction

A food allergy is an immune mediated reaction against an otherwise harmless protein, also known as allergen (1, 2). Cow's milk allergy is one of the most common food allergies in the world which affects about 2.5% of all children (3). Thermal processing of milk is an essential step in the dairy industry to ensure microbiological safety and prolong shelf life (4). However, heat treatments like pasteurization, sterilization and spray drying will affect the structural characteristics of the proteins present in cow's milk, including the whey protein β-lactoglobulin (BLG) which is one of the major allergens present in milk (5–7). Structural modifications like unfolding, aggregation and glycation with lactose could occur during heat treatment, either reducing or enhancing the allergenic potential, i.e., IgE-binding of BLG (8, 9).

After passing through the epithelial cells of the intestine, BLG may be taken up by antigen-presenting cells (APC) of the innate immune system. Of these, macrophages and dendritic cells are considered to be professional APCs (10). They have the ability to capture an allergen into specialized endosomes/lysosomes, and then process it into linear peptides which are expressed on major histocompatibility complex (MHC) molecules on the outer surface, to which a naïve T cell can bind. Although both derived from the same precursor-monocytes, macrophages and dendritic cells exert different functions and possess different functional properties. Macrophages focus on scavenging allergens, manipulating inflammatory responses and recruiting T cells and B cells *in situ*. Dendritic cells are specialized in their ability to preserve the ingested allergens, driving T cell responses following their migration to the secondary lymphoid tissues (11).

To study the immunological response of macrophages and dendritic cells upon exposure to heat-treated BLG, macrophages and dendritic cells derived from THP-1 monocytes were used. This approach would circumvent possible effects of different genetic backgrounds on the cellular responses (12) providing a smaller variability in the read-out, and thereby a clearer picture of the effect of processing of proteins on the cellular response. THP-1 monocytes stimulated by phorbol-12-myristate-13-acetate (PMA) are regarded as macrophage-like cells, showing similarities with primary macrophages in morphology, gene transcription and expression (13–16). There are only few reports describing the differentiation of THP-1 monocytes into dendritic-like cells. THP-1 monocytes, stimulated with PMA together with IL-4, were shown to express typical cellular surface markers and exhibit properties similar to primary immature dendritic cells (17).

By using microarray analysis, the similarities and differences between THP-1 derived macrophages and dendritic cells were analyzed based on their transcriptome. Together with morphology, protein expression levels, and antigen processing capability analyses, their resemblance to primary cells was estimated in comparison with the reported properties of primary cells from literature (18). Differences in uptake capability of THP-1 derived macrophages for differently heat-treated BLG was described previously (19). To obtain an impression of the impact of exposure of THP-1 derived macrophages or dendritic cells to such differently heat-treated BLG on gene expression related to functional properties, the transcription levels of immune related genes and the production of a number of cytokines by THP-1 derived APCs, was analyzed in this study.

## Materials and Methods

### Chemicals

All chemicals were purchased from Sigma Aldrich (St Louis, Missouri, USA) unless otherwise stated.

### THP-1 Cell Culture and Differentiation

Cells from the human monocytic leukemia cell line THP-1 (ATCC, Manassas, Virginia, USA) were differentiated into cells representative of resting macrophages (M0) as described by Chanput et al. (20). Cell suspensions with a concentration of 1 × 10^6^ cells/mL were stimulated using 100 ng/mL phorbol-12-myristate-13-acetate (PMA) in Corning® 24 Well TC-Treated Microplates (Life Sciences, Tewksbury, Massachusetts, USA) with 500 μL volume per well, followed by 2 times washing with the same volume of medium after 48 h and another 48 h of resting. Similarly, using a cell concentration of 0.25 × 10^6^ cells/mL, immature dendritic cells (iDC) were generated after incubating with 20 ng/mL of IL-4 (R&D Systems, Minneapolis, Minnesota, USA) and 20 ng/mL of PMA for 4 days as described by Katayama et al. (17) with the same cell suspension volume and in an identical plate as used for M0. Lipopolysaccharide (LPS) was used to differentiate M0 and iDC, generated with the previous protocol, into LPS-M and mature dendritic cells (mDC) at a concentration of 500 ng/mL and 1 μg/mL, respectively, for 48 h.

### Morphology Measurement of Cells

The morphology of cells in the 24 well-plate was observed by an automated digital inverted microscope Evos FL Auto 2 (Thermo Fisher, Waltham, Massachusetts, USA) using a 40 × objective lens. Scale bars were added to the picture using ImageJ software.

### Sample Preparation and Cell Stimulation

β-Lactoglobulin (BLG) was isolated from raw cow's milk as described in the protocol of De Jongh et al. (21). Lactose was removed by dialysis after which the purity of protein was higher than 94%, confirmed by size exclusion chromatography. In the absence or presence of glucose (molarity 80 times that of BLG), 13 mg/mL BLG was wet-heated (W), high-temperature dry-heated (H) or low-temperature dry-heated (L), centrifuged to remove insoluble material, dialyzed to remove free glucose and the protein concentration measured, as described previously (19). In the annotation of the samples, the first capital (L, H or W) referred to the type of processing, and the indication-glu in the sample name indicated the presence of glucose during the processing step. THP-1 derived M0 and iDC were incubated in Corning® 24 Well TC-Treated Microplates (Life Sciences, Tewksbury, Massachusetts, USA) with 100 μg/mL BLG, L-glu-BLG, H-glu-BLG, W-glu-BLG or the culture medium as a control for 6 h.

### Microarray Analysis

THP-1 derived M0 and iDC were exposed to BLG samples or medium as control in Corning® 24 Well TC-Treated Microplates (Life Sciences, Tewksbury, Massachusetts, USA) for 6 h. RNA of the cultured cells was extracted as described by Tang et al. (22), using 200 μL TRIzol (Invitrogen, Bleiswijk, The Netherlands) per well. Each cell experiment was performed in triplicate with cells from different passage numbers. RNA quality was verified using the RNA 6000 Nano assay on an Agilent 2100 Bioanalyzer (Agilent Technologies, Amstelveen, The Netherlands). Quality approved RNA was labeled and hybridized to the GeneChip® Human Gene 2.1 ST Array in a GeneTitan® platform (Affymetrix, Santa Clara, California, USA) according to standard protocols. The arrays were scanned by the high-resolution scanner of GeneChip Scanner (Affymetrix, Santa Clara, California, USA) and the signal was converted to cell intensity file (.CEL files) using GeneChip Operating Software (GCOS). The raw CEL data was normalized using the Bioconductor packages of MADMAX pipeline (23) and analyzed after data filtering. Final gene transcription levels were quantified by fold change of expression (FC). The FC of each individual gene of M0 or iDC was calculated based on the gene transcription level relative to the level of monocytes, while FC of each individual gene of M0 or iDC incubated with BLG samples was compared with the values for M0 or iDC without incubation, respectively. Genes with an FC for which the absolute value was > 2 and an intensity-based moderated t-statistics (IBMT) raw *q* < 0.05 were considered to be significantly differentially transcribed. The Venn diagrams were generated using Venny 2.1 (https://bioinfogp.cnb.csic.es/tools/venny/).

### Pathway Analysis

Pathway analysis was carried out using the program Ingenuity Pathway Analysis (IPA, Qiagen, Hilden, Germany). For M0 and iDC without BLG incubation and M0 incubated with native and low-temperature dry-heated BLG, only the significantly differentially transcribed genes were uploaded in the software. The IPA core analysis was done based on setting: “Human” in the “Species” category, and “THP-1,” ”Dendritic cells” or “Macrophages” in the category of “Tissue & Cell lines.” Pathways suggested by the software which yielded *p* < 0.001 were considered to be significantly associated with the gene transcription levels of this sample.

### Cell Staining and Flow Cytometry Analysis

THP-1 derived M0 and iDC were cultivated in Corning® 96 Well TC-Treated Microplates (Life Sciences, Tewksbury, Massachusetts, USA) with a volume of 100 μL per well using the same protocol in the previous section on THP-1 cell Culture and Differentiation. The cells were harvested as described previously (19), first washed twice with PBS without Ca^2+^ and Mg^2+^ (Gibco, Thermo Fisher, Waltham, Massachusetts, USA) and incubated with 100 μL of 0.25% Trypsin-EDTA (Gibco, Thermo Fisher, Waltham, Massachusetts, USA) for 5 min, and subsequently centrifuged for 5 min at 450 × g. Cell surface marker staining was performed by first suspending the cell pellets after centrifugation with 100 μL PBS containing 10 μL FcR blocking reagent (Miltenyi Biotec, Auburn, CA) and incubating the cell suspensions at 4°C for 10 min. Then 90 μL PBS containing 1 μL of each detection antibody was added to the cells and incubated for 30 min at 4°C (gently mixed in between once). The following fluorescence-labeled mouse anti-human monoclonal antibodies (mAbs) were used: panel 1 [FITC-conjugated anti-CD209 (clone DCN46), PE-conjugated anti-HLA-DR (clone G46-6), PE-Cy™^7^-conjugated anti-CD83 (clone HB15e) and APC-conjugated anti-CD11c (clone B-ly6)]; panel 2 [FITC-conjugated anti-CD14 (clone M5E2), PE-conjugated anti-CD40 (clone 5C3), PE-Cy™^7^-conjugated anti-CD80 (clone L307.4) and APC-conjugated anti-HLA-ABC (clone G46-2.6)]; and panel 3 [FITC-conjugated anti-CD63 (clone H5C6) and APC-conjugated anti-CD81 (clone JS-81)] all from BD Biosciences (Franklin Lakes, New Jersey, USA). After staining, 1 mL PBS was added to the cells followed by centrifugation for 5 min at 450 × g. The cell pellet was resuspended in 150 μL PBS with Ca^2+^ and Mg^2+^ and the mean fluorescence intensity (MFI) of each fluorescence signal was analyzed for four-color immunofluorescence with an Accuri C6 flow cytometry set to compensate for overflow (BD Biosciences, Franklin Lakes, New Jersey, USA) of at least 5,000 cells for each sample using BD Accuri C6 software. The MFI of unstained cells was subtracted from each value after correcting the overflow of each fluorescence.

### Cytokine Secretion Measurement

Supernatant of THP-1 derived M0 and iDC was collected following 16 h stimulation with medium or 100 μg/mL sample. The cytokine secretion measurement was done with the ELISA Deluxe Set Human IL-8, IL10, IL-6, TNF-α, CCL20, and IL-1β (Biolegend, San Diego, California, United States) following the manufacturer's protocol. The absorbance was measured with Infinite® 200 PRO NanoQuant (Tecan, Männedorf, Switzerland).

### Assessing the Antigen Processing Capacity of Cells Using DQ-Ovalbumin (DQ-OVA)

DQ-OVA is a self-quenched conjugate of ovalbumin which is strongly labeled with BODIPY dyes. It will exhibit bright green fluorescence upon proteolytic degradation into single, dye-labeled peptides. THP-1 derived M0 or iDC cells in Corning® 96 Well TC-Treated Microplates (Life Sciences, Tewksbury, Massachusetts, USA) with a volume of 100 μL per well using the same protocol as in the previous section on THP-1 cell Culture and Differentiation were incubated with medium containing 10 ug/mL DQ-OVA (Life Sciences, Tewksbury, Massachusetts, USA) for 1 h. After that, cells were washed twice with PBS without Ca^2+^ and Mg^2+^ (Gibco, Thermo Fisher, Waltham, Massachusetts, USA) and incubated with 100 μL of 0.25 % Trypsin-EDTA (Gibco, Thermo Fisher, Waltham, Massachusetts, USA) for 5 min. The cell suspension in Trypsin-EDTA was centrifuged for 5 min at 450 × g, after which the cell pellets were resuspended in 150 μL PBS with Ca^2+^ and Mg^2+^. The mean fluorescence intensity (MFI) of the FITC signal was recorded by flow cytometry of at least 5,000 cells for each sample using BD Accuri C6 software.

### Statistics

The statistical analysis of microarray data was performed as described in the previous section on Microarray Analysis. The statistical analysis of other data was done using Prism 6 software or R studio (package “agricolae”), considering *p* < 0.05 to be significant.

## Results

### THP-1 Monocyte-Derived Macrophage- and Dendritic-Like Cells Differed in Morphology

**Figure 1:**
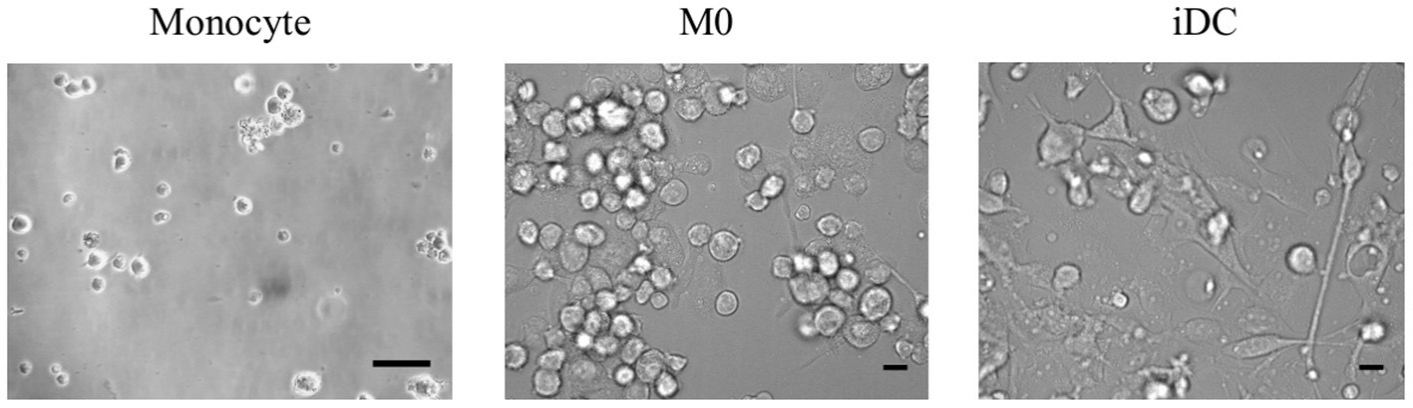
The morphologies of THP-1 monocytes and their derived macrophage-like cells at resting stage (M0) and immature dendritic-like cells (iDC) can be distinguished from each other. THP-1 monocytes were non-treated or differentiated into M0 or iDC in a 24 well-plate and analyzed under the microscope. Scale bar is 20 μm. Representative pictures of repeated differentiation experiments were shown.

To build genetically homogenous immune cell models, we used the THP-1 monocytic cell line to obtain macrophage- and dendritic-like cells. After 2 days of culturing in medium supplemented with 100 ng/mL of PMA followed by 2 days of rest or 4 days with 20 ng/mL of PMA and 20 ng/mL of IL-4, THP-1 monocytes differentiated into macrophage-like cells in the resting stage (M0) or immature dendritic-like cells (iDC), respectively. We first compared the morphology of M0 and iDC to their precursor monocytes. Morphology of both induced cell types demonstrated an increase in size, while M0 maintained their globosity, and iDC showed an elongated shape (Figure 1). In addition, both induced cell types were adherent in contrast to the monocytes.

### Gene Transcription, Upstream Regulation, and Pathway Analysis of M0 and iDC Showed Different Profiles

**Figure 2:**
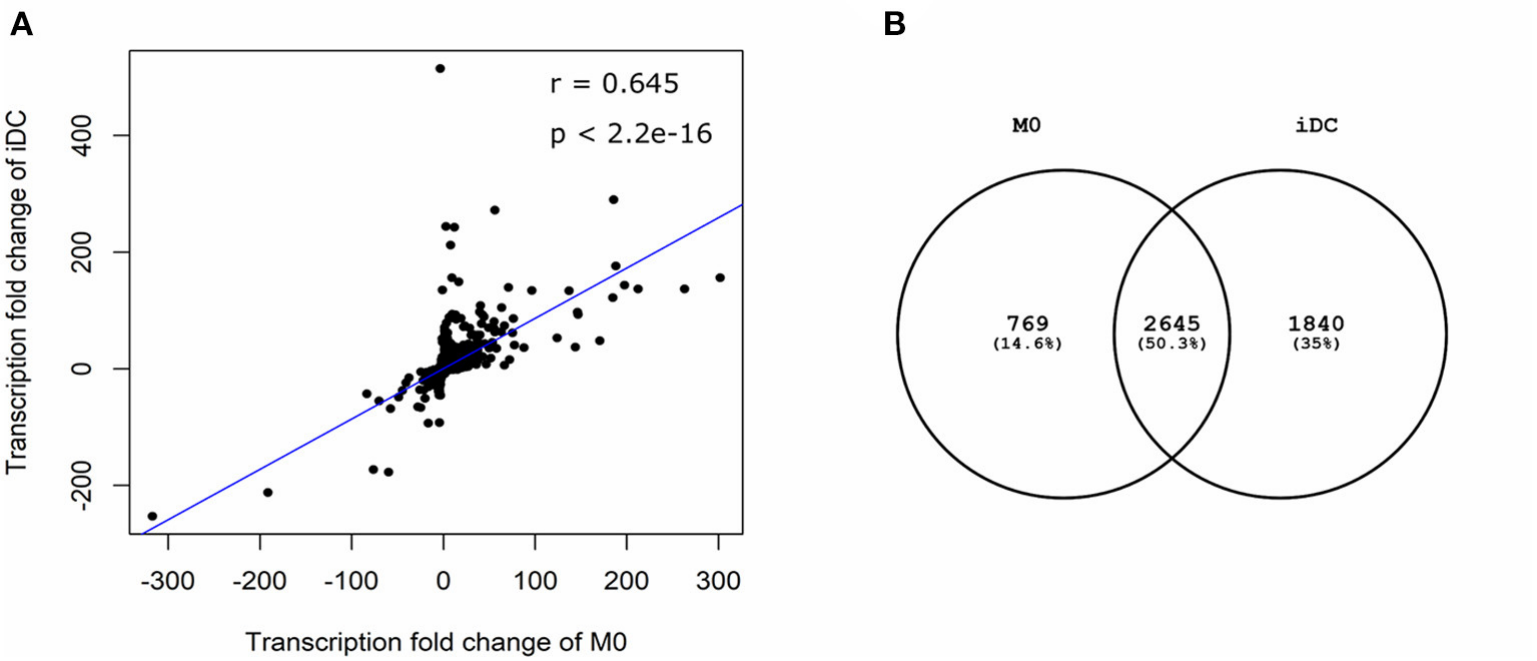
Differential gene transcription in THP-1 derived macrophage- (M0) and dendritic-like cells (iDC) showed similarities and differences. THP-1 monocytes were non-treated or differentiated into M0 and iDC and the transcriptome was analyzed using microarray. Microarray analysis provided data on the transcriptome which was expressed as average fold change (FC) between differentiated cells vs. monocytes of 3 independent experimental replicates. Genes with an absolute value of FC > 2 and *q* value < 0.05 in M0 or iDC when compared to non-treated monocytes were considered to be significant. A scatter plot **(A)** showed the correlation between significantly differentially expressed genes in M0 and iDC. The Pearson correlation coefficient (*r*) and two-tailed *p*-value were analyzed using R studio. The Venn diagram **(B)** showed the numbers of shared or exclusive significantly differentially expressed genes in M0 and iDC.

In order to comprehensively analyse and compare M0 and iDC, mRNA was extracted and gene transcription was analyzed. The gene transcription of M0 and iDC was compared qualitatively and quantitatively to that of monocytes to obtain an indication of up- or down-regulation of specific macrophage and dendric cell related genes. In total, 5,307 genes were found to be significantly differentially transcribed in at least one of the cell types out of more than 25,000 tested genes. These genes revealed a moderate correlation (Figure 2A; *r* = 0.645, *p* < 0.001) between M0 and iDC in a scatter plot. Moreover, half of the significantly differentially transcribed genes were shared by both cell types, but these cell types also showed many uniquely transcribed genes (Figure 2B). These results were further analyzed based on the top 25 genes with the highest FC for M0 (Supplementary Table 1) and for iDC (Supplementary Table 2). In general, the fold changes in transcription levels of iDC were much higher than for M0. Supplementary Table 1 shows that the transcription levels of CYBB, IL7R, CD180, WIPF3, KCNMA1, MMP2, TLR7, ZC3H12D and CHODL were higher in M0 and these genes are related to antigen uptake, innate immunity and cell signaling (24–27). Supplementary Table 1 also shows increased transcription levels of ENDRA, S1PR1, CHRM3, C3AR1 in M0 which are related to G proteins and coupled receptors (28–31). Supplementary Table 2 contains four SERPINB related genes with increased transcription in iDC, which might be due to the IL-4 used for cell stimulation (32, 33). Furthermore, IPA indicated AQP9, CCL22, INHBE, CD274, MMP7, and VCAM1 (Supplementary Table 2) to be related to immunological responses (34–39), EHF, SCIMP, NR4A3, and ALDH1A2 to be related to dendritic cells (40–43), and GJB2 and VCAM1 to be related to cell adhesion (38, 44).

**Figure 3:**
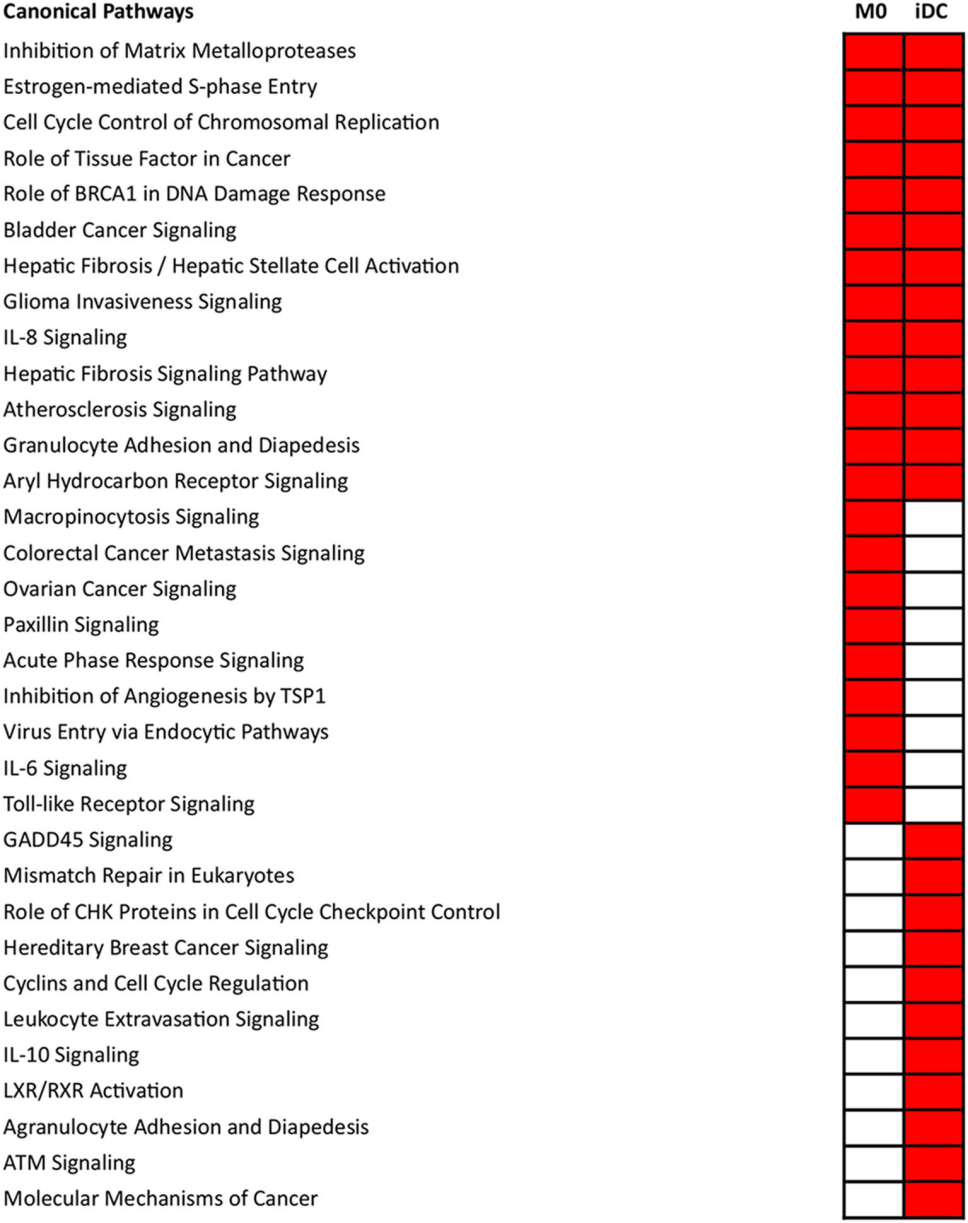
Significantly associated canonical pathways in THP-1 derived macrophage- (M0) and dendritic-like cells (iDC) differed from each other. THP-1 monocytes were non-treated or differentiated into M0 or iDC. Microarray analysis provided data on the transcriptome which was expressed as average fold change between differentiated cells vs. monocytes of 3 independent experimental replicates. Then, the transcriptomes were imported into IPA for canonical pathway analysis. Canonical pathways with a *p* < 0.001 were selected, labeled in red and listed separately according to the categories: present in both M0 and iDC, only M0 and only iDC. In each category, the pathways were listed from top to bottom following the *p*-value difference between M0 and iDC from large to small.

The microarray data were analyzed in a more comprehensive manner with Ingenuity Pathway Analysis (IPA) based on the significantly differentially transcribed genes for M0 and iDC. Pathway analysis is based on gene transcription of proteins that are empirically shown to interact in cellular pathways, providing evidence that a specific pathway might be activated or inhibited. Pathway analysis yielded significantly associated canonical pathways (*p* < 0.001) which are listed in Figure 3. Associated with both cell types were, amongst others, the pathways “Inhibition of Matrix Metalloproteases” and “Atherosclerosis Signaling and Aryl Hydrocarbon Receptor Signaling,” which are related to cell differentiation or proliferation and the pathways “IL-8 Signaling” and “Granulocyte Adhesion and Diapedesis,” which are related to inflammation and immune response. There were 9 pathways significantly associated with M0, among which “Macropinocytosis Signaling” and “Virus Entry via Endocytic Pathways” (both uptake related), “Paxillin Signaling” (cell adhesion and morphology related), “Acute Phase Response Signaling,” “IL-6 Signaling” and “Toll-like Receptor Signaling” (both proinflammatory and cytokine related). Eleven pathways were significantly associated with iDC, among which “Leukocyte Extravasation Signaling,” “IL-10 Signaling,” and “Agranulocyte Adhesion and Diapedesis” are all related to immune responses. Next to pathway analysis, IPA also predicted activation or inhibition of upstream regulators. This is done by comparing the microarray data to known gene transcription profiles linked to individual upstream regulators, which provides insights into potential activation or inhibition. As shown in Supplementary Table 3, upstream regulators ECSIT (intermediate in TLR Pathway) (45), CD40LG, TNF-α, TLR4, CD40, IL17A, IL6, and TNFRSF1A were found to be activated in both M0 and iDC.

### Surface Marker Expression, Cytokine Production, and Antigen Processing of M0 and iDC

**Figure 4:**
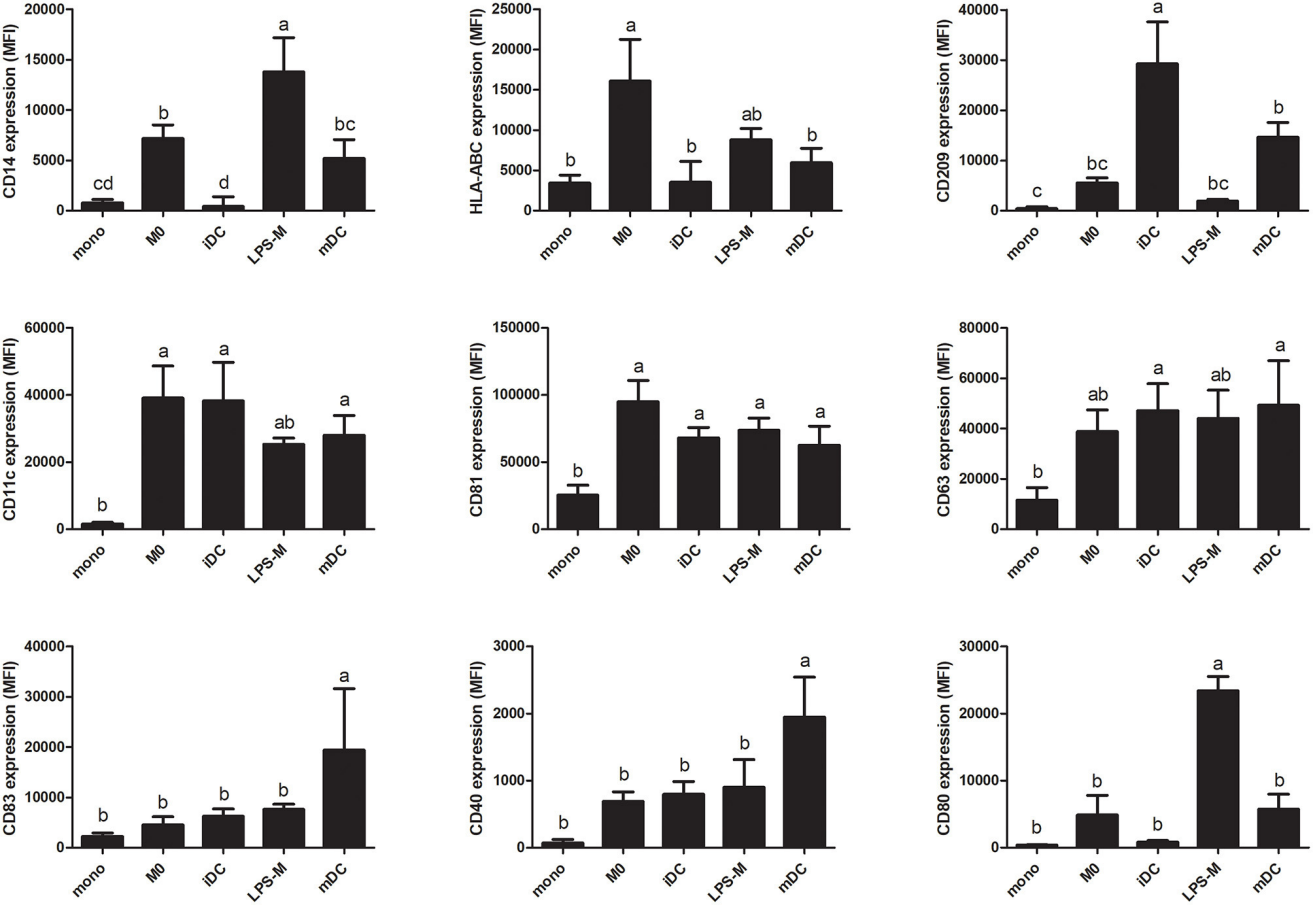
THP-1-derived macrophage- (M0) and dendritic-like cells (iDC) and their LPS-stimulated forms LPS-M and mDC showed different surface marker profiles compared to monocytes. THP-1 monocytes were non-treated or differentiated into M0 and iDC and/or stimulated with LPS yielding LPS-M and mDC, respectively. Expression of surface markers was analyzed through staining with fluorescent-labeled antibodies and MFI was measured by flow cytometry. The results represent the mean MFI ± SD of 5 independent experiments. The statistical differences were calculated with Fishers Least Significant Difference (LSD) test and bars with different letters represent significant differences (*p* < 0.05).

To further characterize the two different cell types generated from THP-1 monocytes, we measured their cell surface marker expression and secretion of cytokines. In addition, to compare cells in an inflammatory state, we challenged them with LPS, resulting in LPS-stimulated macrophages (LPS-M) and mature dendritic cells (mDC). As shown in Figure 4, expression of circulating monocyte-derived macrophage differentiation markers CD14 and HLA-ABC (46, 47) were significantly higher in M0 compared to monocytes or iDC, while the expression of circulating monocyte-derived dendritic cell marker and T cell activator CD209 (48) was significantly higher in iDC compared to monocytes or M0. Expression levels of primary APC surface markers CD11c and CD81 (49, 50) were similar in M0 and iDC and both significantly higher than in monocytes. Furthermore, CD63 which was reportedly expressed on circulating monocyte-derived immature dendric cells (51), showed significantly higher expression in iDC but not in M0 compared to monocytes. After LPS stimulation, expression of CD83 and CD40, which are related to dendritic cell maturation and interaction with T and B cells (52), were significantly higher in mDC than iDC or LPS-M. CD209 was significantly decreased in mDC compared to iDC. On the other hand, cell surface expression levels of CD14 and CD80 (activated circulating monocyte-derived macrophage marker) (53) were significantly higher on LPS-M than on both M0 and mDC.

**Figure 5:**
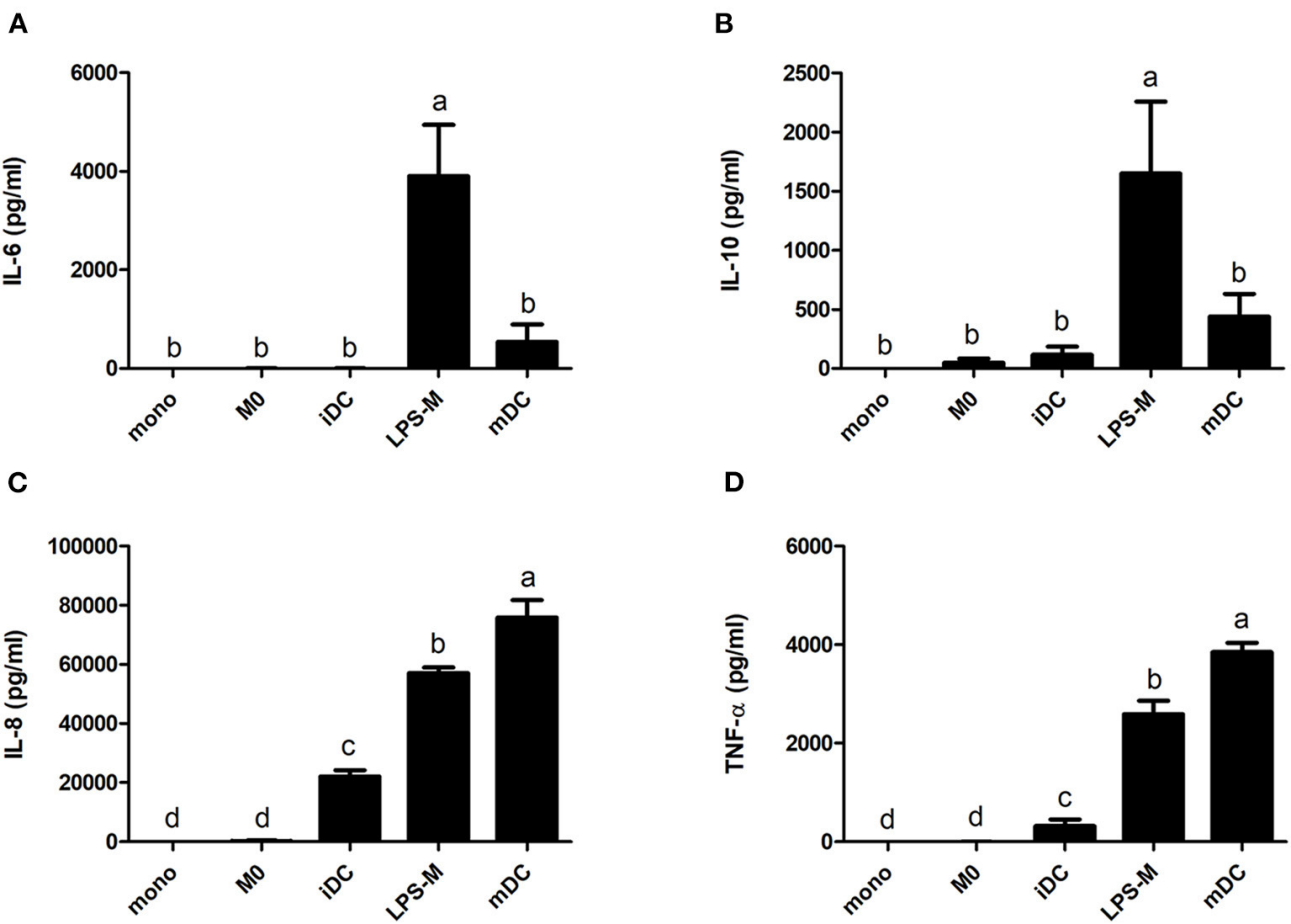
LPS-mediated activation resulted in significantly different increases of cytokine production for THP-1-derived macrophage- (M0) and dendritic-like cells (iDC). THP-1 monocytes were non-treated or differentiated into M0 and iDC and stimulated with LPS. The supernatant of the cell cultures was collected, and the cytokines levels of IL-6 **(A)**, IL-10 **(B)**, IL-8 **(C)**, and TNF-α **(D)** were determined using ELISA. The results represent mean values ± SD of 2–4 independent experimental measurements. The statistical differences were calculated with Fishers Least Significant Difference (LSD) test and bars with different letters represent significant differences (*p* < 0.05).

The secretion of cytokines also showed significant differences between M0 and iDC, and their corresponding cell types after LPS stimulation (Figure 5). Of note, none of the tested cytokines (i.e. IL-6, IL-10, IL-8, TNF-α, and IL-12) reached threshold detection levels in the monocyte culture (data not shown). We also did not observe a significant increase in IL-6 or IL-10 secretion upon differentiation to M0 or iDC. However, the secretion levels of IL-8 and TNF-α (proinflammatory cytokines) in iDC were significantly higher than in M0 (avg. 22.0 vs. 0.2 and 0.3 vs. 0 ng/mL, respectively). LPS stimulation led to higher cytokine production for both stimulated cell types compared to their corresponding non-stimulated cells for all tested cytokines. The significantly higher secretion levels of IL-8 and TNFα in iDC compared to M0 were maintained in their LPS-stimulated cells with a general higher production level (avg. 75.8 vs. 57.1 and 3.9 vs. 2.2 ng/mL). On the contrary, the production of IL-6 and IL-10 in LPS-M became significantly higher than in mDC (avg. 1.7 vs. 0.4 and 3.9 vs. 0.5 ng/mL). IL-12 production was not detected in any of the cell types or differentiation stages.

**Figure 6:**
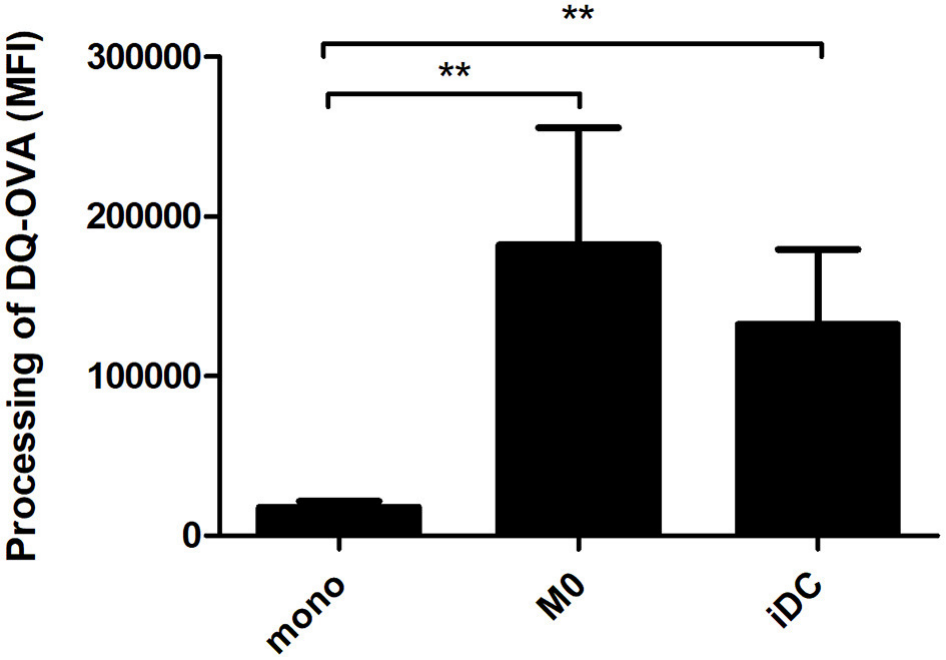
THP-1-derived macrophage- (M0) and dendritic-like cells (iDC) revealed a significantly increased capacity to process antigens compared to monocytes. Proteolytic cleavage of fluorophores from DQ-OVA (initially auto-quenching due to heavily labeling) result in fluorescent signals. THP-1 monocytes were non-treated or differentiated into M0 and iDC and incubated with DQ-OVA for 2 h, after which the florescence signal was measured by flow cytometry. The results represent the average MFI ± SD of 5 independent experimental measurements. The statistical differences between any two data set were calculated with two-tailed unpaired *T*-test, **p < 0.01.

A key hallmark of APCs is the internalization and processing of antigens for subsequent presentation to adaptive immune cells. Using DQ-OVA, we tested the antigen uptake and processing capacity of M0 and iDC. Comparing the numbers of cells that were able to process DQ-OVA, there were no significant differences between the phenotypes (Supplementary Figure 1). Comparing the MFI-values, both M0 and iDC showed significantly increased antigen processing capacity compared to monocytes, but no significant difference was observed between M0 and iDC (Figure 6).

### M0 and iDC Responded Differentially to Exposure to Native or Heat-Treated BLG Based on Global Gene Transcription Profiles

Upon establishing the differences between M0 and iDC, and their similarities with primary monocyte derived macrophages and dendritic cells, the THP-1 derived M0 and iDC model was employed to study the impact of processed BLG samples. M0 and iDC were exposed to native, wet-heated, high- and low-temperature dry-heated BLG in the presence of glucose (BLG, W-glu-BLG, H-glu-BLG, and L-glu-BLG, respectively), after which gene transcription levels were analyzed using microarray and compared to non-treated M0 and iDC.

**Figure 7:**
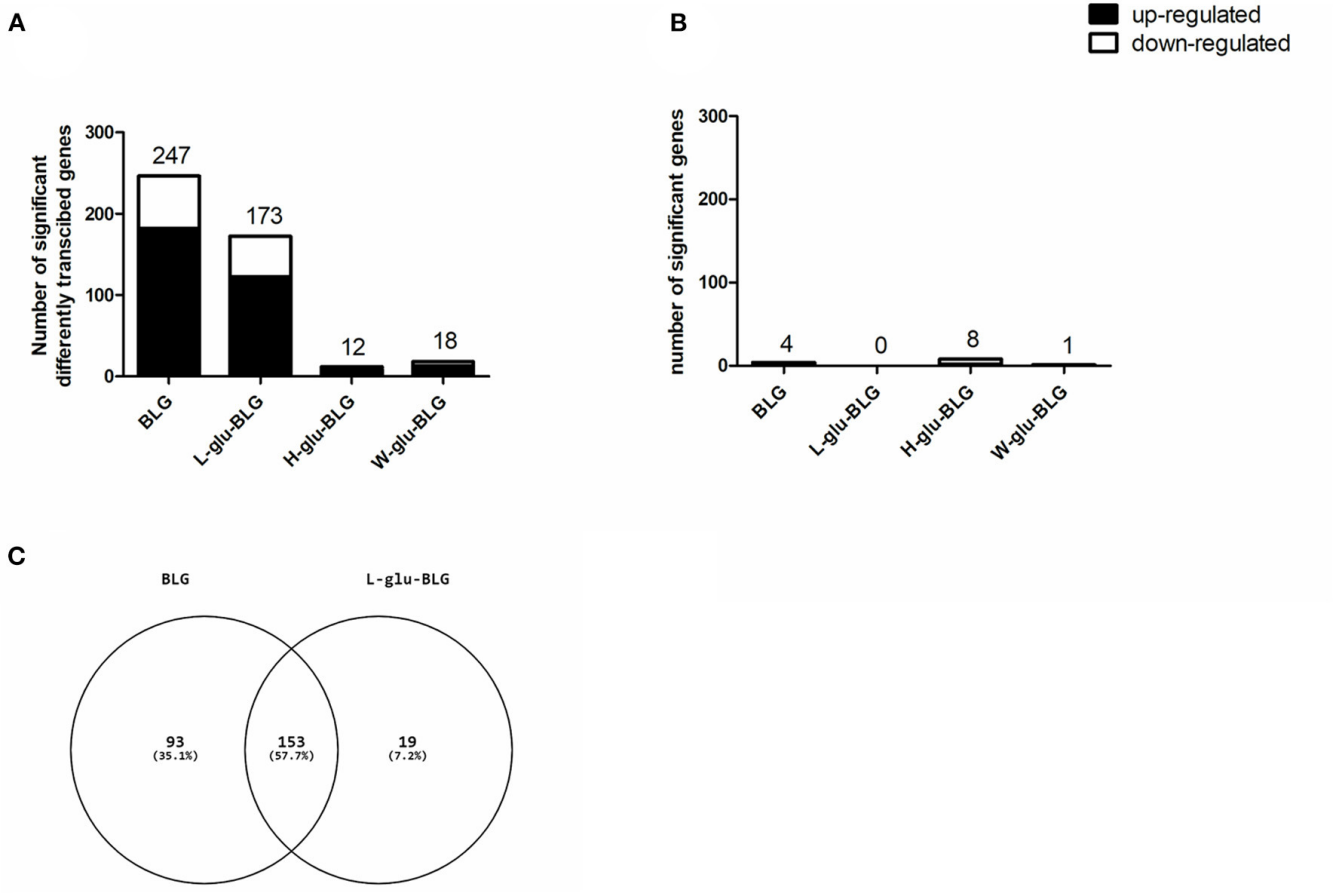
After exposure to BLG samples, THP-1 derived dendritic-like cells (iDC) hardly showed a response in gene transcription profiles if compared to medium control, while the response of macrophage-like cells (M0) mainly occurred in response to native and L-glu-BLG. THP-1 monocytes were differentiated into M0 and iDCs and incubated with medium (non-treated) or different BLG samples (native, L-glu-BLG, H-glu-BLG, or W-glu-BLG) for 6 h. Microarray analysis provided data on the transcriptome which was expressed as average fold change between treated vs. non-treated cells of 3 independent experimental replicates. The bar chart indicates the number of significantly (with fold change absolute value > 2 and *q* value < 0.05) up or down regulated genes for M0 **(A)** and iDC **(B)** treated with native or processed BLG. In a Venn diagram the numbers of significantly transcribed genes which are shared or exclusive for M0 incubated with BLG or L-glu-BLG were shown **(C)**.

In general, there were more upregulated than downregulated genes in either cell type treated by either sample type. In M0 treated with BLG and L-glu-BLG, the highest total number of significantly differentially transcribed genes was found (247 and 173, respectively; Figure 7A). Figure 7C showed that most of the significantly differentially transcribed genes in M0 incubated with L-glu-BLG were also significantly differently transcribed in M0 incubated with BLG. In M0 treated with H-glu-BLG or W-glu-BLG and iDC treated with any sample, the number of significantly differentially transcribed genes was below 20 (Figures 7A,B), for which details can be found in Supplementary Tables 4, 5.

### Immunity Related Pathways and Upstream Regulators Were Significantly Associated With and Activated in M0 Treated With Native or Low-Temperature Dry-Heated BLG

**Figure 8:**
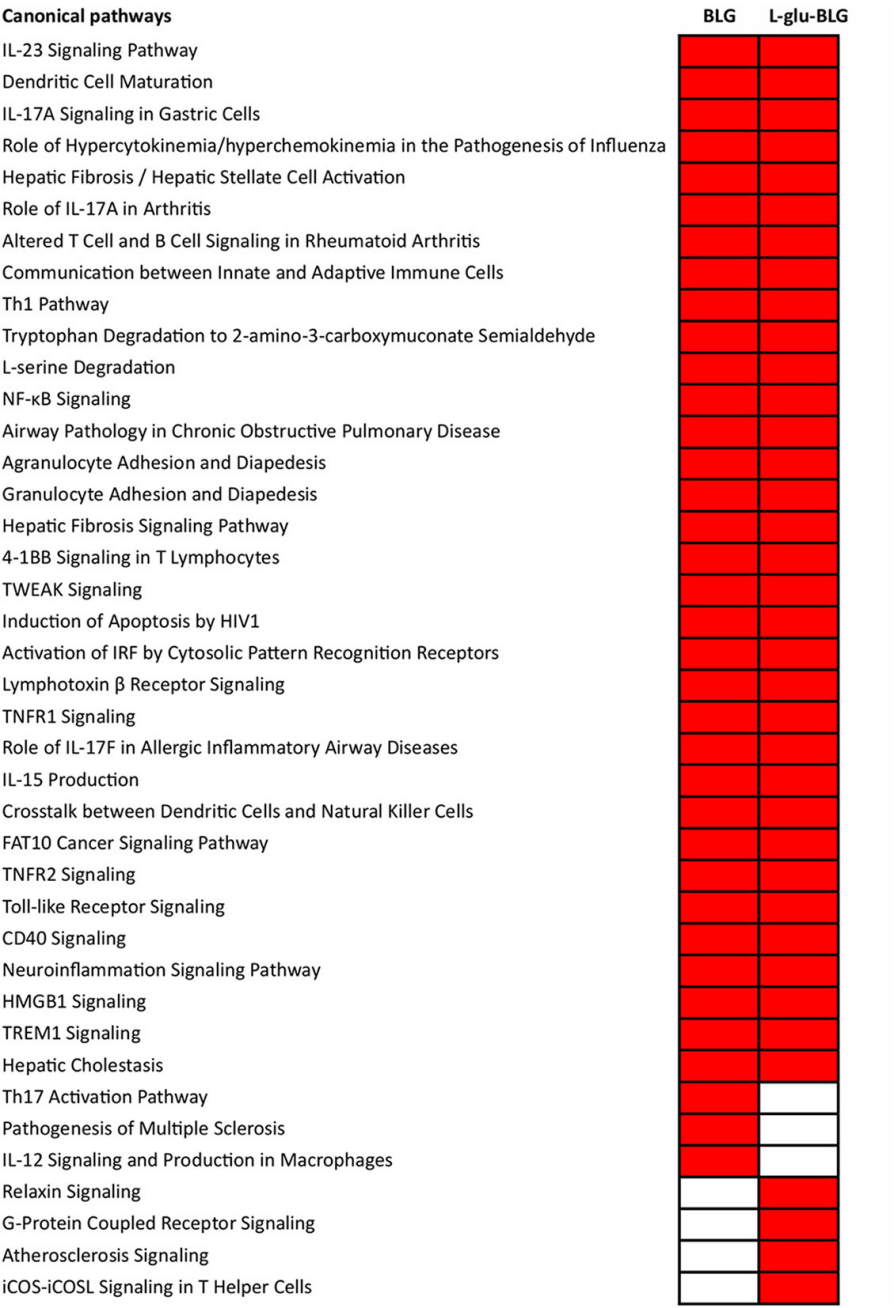
Significantly associated canonical pathways in THP-1 derived macrophage-like cells (M0) incubated with BLG and L-glu-BLG were mostly related to immune responses. THP-1 monocytes were differentiated into M0 cells and were incubated with medium (non-treated), native or L-glu-BLG for 6 h. Microarray analysis provided data on the transcriptome which was expressed as average fold change between treated vs. non-treated cells of 3 independent experimental replicates. Then, the transcriptomes were imported into IPA for canonical pathway analysis. Canonical pathways with a *p* < 0.001 were selected, labeled in red and listed separately according to the categories: present in both native and L-glu-BLG treated M0, only native treated M0 and only L-glu-BLG treated M0. In each category, the pathways were listed from top to bottom following the *p*-value difference between native and L-glu-BLG treated M0 from large to small.

A more comprehensive analysis of gene transcription profiles of M0 treated with native or low-temperature dry-heated BLG was performed with IPA. Analysis of significantly associated canonical pathways revealed that both BLG and L-glu-BLG induced immune related pathways in M0 (Figure 8). Inflammatory-related pathways were significantly associated with M0 treated with BLG and L-glu-BLG, including “Th1 Pathway,” “NF-κB Signaling,” “TNFR1 Signaling,” “TWEAK Signaling,” “Activation of IRF by Cytosolic Pattern Recognition Receptors,” “TNFR1 Signaling,” “Toll-like Receptor Signaling,” “CD40 Signaling,” “HMGB1 Signaling,” and “TREM1 Signaling.” Additionally, pathways such as the “IL-23 Signaling Pathway” (Th17 maintenance and expansion), “Communication between Innate and Adaptive Immune Cells,” “Agranulocyte Adhesion and Diapedesis,” “Granulocyte Adhesion and Diapedesis” and “IL-15 Production” (regulatory of natural killer cells and T cells), which are all related to adaptive immunity signaling, were also significantly associated with M0 treated with BLG and L-glu-BLG. Exposure of M0 to BLG, but not L-glu-BLG, showed a significant association with the “Th17 Activation Pathway” and “IL-12 Signaling and Production in Macrophages.” On the contrary, several B cell related and Th2 pathways were only significantly associated with M0 incubated with L-glu-BLG.

As shown in Supplementary Table 6, in M0 treated with native BLG, innate immune related regulators ECSIT, IFNG (IFNγ), TLR4, IL27, TLR2, TNF (TNF-α), and IL-1β were significantly activated while LY6E, which has immunosuppressive effects (54), was inhibited. There was no clear difference in the type of activated or inhibited upstream regulators in M0 incubated with L-glu-BLG compared to BLG, but the activation levels (based on z-scores) were weaker in the former condition.

### Significantly Higher Transcription and Secretion Levels for Specific Cytokines Could Be Found in Native and Low-Temperature Dry-Heated BLG Treated M0

**Figure 9:**
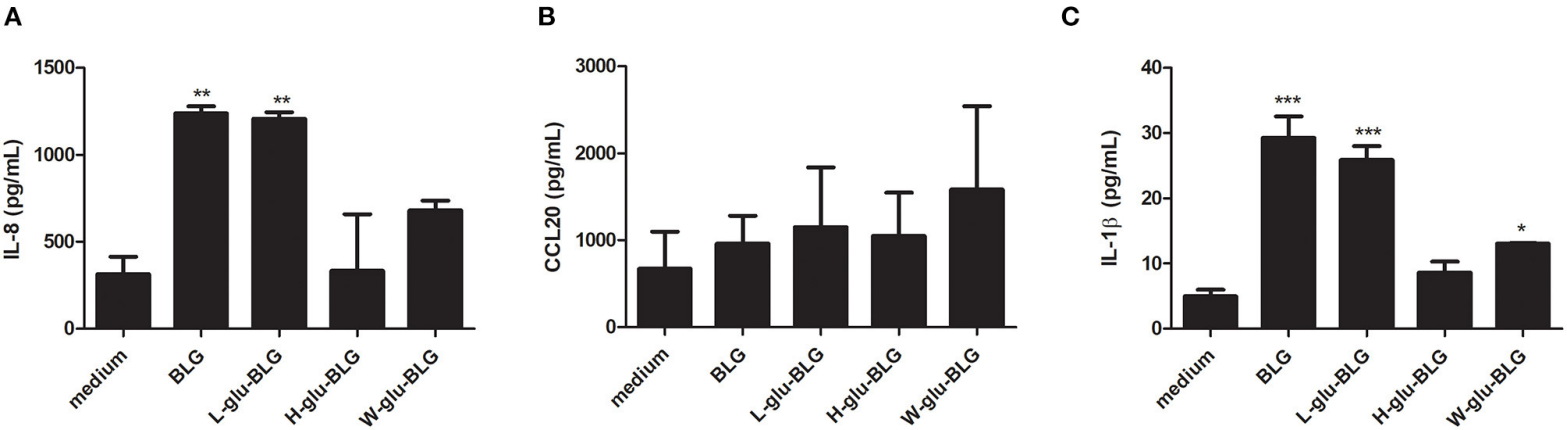
Cytokine response of THP-1 derived dendritic-like cells (iDC) upon exposure to native or heat-treated BLG varies. BLG was untreated or heated (W, H, or L) in the presence of glucose and incubated with iDC. The cytokine secretion of IL-8 **(A)**, CCL20 **(B)**, and IL-1β **(C)** in the supernatant was analyzed with ELISA. The results represent mean values ± SD of 3 independent experimental measurements. The statistical differences were calculated with Dunnett's Test compared to medium control: *p < 0.05; **p < 0.01; ***p < 0.001.

Similar as for establishing the M0 and iDC cell models, cytokine and chemokine analysis was performed to further characterize M0 responses to native and L-glu-BLG. From the gene transcription data, cytokine genes with a significantly differential transcription level in M0 following incubation with any BLG sample are presented in Table 1 (there were no significantly differentially transcribed cytokine gene for any of the iDC stimulations). In fact, significantly differentially transcribed cytokine or chemokine genes were found only in M0 incubated with BLG or L-glu-BLG with transcription levels being slightly higher in the former. Significant increases in transcription levels when compared to medium stimulated M0 were found for IL23A, IL32, CCL1, CCL4, CCL5, CCL19, CCL22, CXCL3, CXCL8, CXCL10, and CXCL11 following M0 stimulation with native BLG and/or L-glu-BLG. The transcriptional data was verified by protein detection for IL-8 (CXCL8), CCL20 and IL-1β. M0 incubated with native or heat-treated BLG samples showed a significantly higher secretion of all cytokines after treatment with native BLG and L-glu-BLG compared to other BLG samples (55). In iDC, BLG and L-glu-BLG similarly induced a significantly increased secretion of IL-8 and IL-1β, but not CCL20 (Figure 9).

**Table 1:** Transcription fold change of cytokine genes in M0 incubated with BLG samples.

**Cytokine**	**BLG**	**L-glu-BLG**	**H-glu-BLG**	**W-glu-BLG**
IL23A	2.3^*^	1.6	1.2	1.0
IL32	3.4^*^	3.8^*^	1.8	2.1
CCL1	4.9^*^	2.6^*^	1.7	1.4
CCL4	4.5^*^	3.3	1.3	1.6
CCL5	2.2^*^	1.8^*^	1.1	1.2
CCL19	4.9^*^	4.3^*^	1.3	1.5
CCL22	3.7^*^	2.7^*^	1.6	1.6
CXCL3	3.5^*^	3.3	−1.2	1.1
CXCL8	14.0^*^	9.0	2.2	3.5
CXCL10	3.8^*^	5.7^*^	1.4	2.1
CXCL11	2.1^*^	1.8	1.3	1.2

*The values indicate fold changes of the transcription of THP-1 derived M0 exposed to different BLG samples compared to their control (exposed to culture medium) of N = 3 experiments*,

* *q < 0.05*.

## Discussion

The differentiation of THP-1 monocytes into macrophage-like cells (M0) by PMA is a common method, whereas studies about the differentiation into dendritic-like cells are relatively limited (56). Based on the protocol of Katayama et al. (17), we generated THP-1 derived immature dendritic-like cells (iDC) using PMA and IL-4. The morphology, transcription profile, expression and secretion levels of surface markers and cytokines and chemokines, and antigen processing capability of THP-1 derived M0 and iDC were determined, and these were compared to their progenitor monocytes. Although PMA was used to derive both cell types, the lower concentration of PMA and the addition of IL-4 induced dendritic-like cells as a clearly different cell type from macrophages and monocytes. The difference of M0 and iDC from their progenitor THP-1 monocytes is clear from their morphology (Figure 1), as well as the vast number of significantly transcribed genes compared to monocytes (Figure 2). There were in total 5,254 genes significantly differentially transcribed in M0 and iDC compared to monocytes, of which about half were shared by both cell types. From the canonical pathway analysis in Figure 3, both M0 and iDC showed significant association with cell differentiation and immune response related pathways, indicating their differentiated cell identities and essential role in immune responses. Their antigen processing capabilities were illustrated by the enhanced proteolytic cleavage of DQ-OVA when compared to monocytes, as shown in Figure 6.

The difference between iDC and M0 was also obvious from their morphology, with a typical circular shape and the presence of dendrites for iDC (Figure 1). When looking at the significantly differentially transcribed genes compared to monocytes, the correlation between the fold change of M0 and iDC is moderate (Figure 2A), but with many uniquely differentially transcribed genes for each cell type, suggesting clear phenotypic differences. The fact that iDC had a higher number of significantly differentially transcribed genes than M0 might be caused by stronger activation of upstream regulators (TNF-α, TLR4, TREM1, and TSLP) and secretion of IL-8 and TNF-α compared to M0 (Supplementary Table 3 and Figure 5). TNF-α and TLR4 play essential roles in activating T cells (57, 58). This coincided with the significantly associated pathways Leukocyte Extravasation Signaling and IL-10 Signaling (Figure 3). The C-type lectin-like receptor CD209 (DC-SIGN), which was significantly stronger expressed in iDC than in THP-1 monocytes and M0, is involved in dendritic cell-T cell interaction (59). These observations indicate the additional T cell signaling function of iDC next to their role in phagocytosis and other immune regulatory functions as APCs (60). On the other hand, M0 showed exclusive and significant associations with uptake and proinflammatory related pathways (Figure 3), which is in line with the more prominent role of M0 in inducing phagocytosis and inflammation compared to iDC. Expression of CD14 was significantly higher in M0, which is reported to be a pattern recognition receptor for LPS and related to the innate immune response of macrophages (61). LPS-stimulated M0 secreted significantly higher amounts of IL-6 and IL-10 (Figure 5), which is in line with other studies (62, 63). The transcription levels of the surface markers (Supplementary Table 7) corroborated the outcomes of the surface marker expression for both M0 and iDC. Overall, our THP-1 derived M0 and iDC could be distinguished from each other and showed clearly differing properties. Moreover, they successfully represented the primary macrophages and dendritic cells by showing antigen uptake, processing and inflammatory response by M0, and the function of T cell stimulation and signaling between the innate and adaptive immune system by iDC.

Although iDC had more significantly differentially transcribed genes and higher transcription levels compared to monocytes than M0, its response to BLG samples was much weaker than M0 in both transcription and cytokine secretion levels (Figures 7, 9). BLG was reported to have limited immunomodulatory effects when incubated with murine DC (64), whereas its immune effect on macrophages has not been clearly studied yet. As shown in Table 1 and Figure 9, incubation of M0 with native or L-glu-BLG induced increased gene transcription and protein secretion of cytokines when compared to W-glu-BLG or H-glu-BLG. The latter two samples were also reported to show the strongest difference from native BLG in terms of structure and uptake by M0 as described in our previous study (19). Thus, the lower cytokine secretion levels of the M0 treated by W-glu-BLG or H-glu-BLG compared to native BLG may be related to their severe structural change after heat processing.

The inflammatory pathways and activation of innate immunity related upstream regulators in M0 that were significantly associated with incubation with BLG and L-glu-BLG (Figure 8 and Supplementary Table 6) appear to indicate a pro-inflammatory effect of BLG. A considerable number of adaptive immunity and T cell signaling pathways were activated, which is not surprising as other studies also demonstrated that macrophages have the ability to regulate adaptive immune responses and even directly contact T cells (65, 66). The pro-inflammatory effect of BLG and L-glu-BLG could possibly be linked with the significant association of T helper 1 (Th1) Pathways (67). In addition to the Th1 response, the response of M0 to incubation with the BLG sample also seemed to impact Th17 cells, as suggested by the significant association with IL-23, Th17, and several IL-17 related pathways. There was no clear difference in the activation of upstream regulators by M0 incubated with BLG or L-glu-BLG, and they also shared most of the significantly associated pathways. In a study, where human primary monocyte-derived dendritic cells were used and the cytokine profile of CD4^+^ T cells, co-cultured with these dendritic cells, was measured, exposure to native ovalbumin was also associated with Th1 cytokine induction (IFN-γ) (68). Endocytic uptake is the crucial and fundamental step in inducing further responses by APCs (69). As indicated by our results, M0 incubated with BLG samples showed stronger transcriptional responses than iDC. Wet-heated BLG, which has undergone the severest physicochemical modifications and also showed the highest uptake among all tested samples (19), did not cause the strongest transcriptional response by innate immune cells. There is thus no clear relationship between the uptake efficiency in M0 and the transcription-inducing effects of BLG. Although the underlying mechanisms are unclear, the data show that less modified BLG leads to a more extensive transcriptional response, indicating that extensive heating destructs immunogenic structures on BLG. Glycation, as occurring at low-temperature dry-heated conditions, might change the immunogenicity of BLG toward a Th2 differentiation enhancing direction which is related to chronic inflammatory disease and allergy (70).

In conclusion, dendritic-like cells were generated successfully from THP-1 monocytes, which clearly differed from both their progenitor and M0. In addition, a high level of uptake of processed BLG by APCs was shown to not be linked to a high level of gene transcription and cytokine production. It should be noted that this work is based on *in vitro* experiments, the result of which still need to be further verified *in vivo*, as functional responses of immune cells, rather than uptake or gene transcription alone, are required to identify the immunogenicity of proteins before and after processing-induced changes.

## Data Availability Statement

The datasets generated for this study can be found in the article/Supplementary Material.

## Author Contributions

YD acquired and analyzed all data. CG, EB, and A-JD contributed to the data analysis. YD, CG, KH, and HW participated in the design and interpretation of the reported experiments and results. YD drafted and edited the manuscript. CG, KH, and HW revised and edited the manuscript. All authors read and approved the final manuscript.

## Conflict of Interest

The authors declare that the research was conducted in the absence of any commercial or financial relationships that could be construed as a potential conflict of interest.
