# Supplementary material for: A THP-1 Cell Line-Based Exploration of Immune Responses Toward Heat-Treated BLG
Source: Front Nutr. 2021 Jan 13;7:612397. doi: 10.3389/fnut.2020.612397 (PMC7838438; doi:10.3389/fnut.2020.612397)
Supplement: Supplementary file 1 [file Table_1.docx]

**Table S1.** Top 25 genes that appear typical for M0 when compared to iDC

| **Gene name** | **M0** | **iDC** | **Gene Ontology Description** |
| --- | --- | --- | --- |
| CYBB | 15.1* | -1.6 | inflammatory response, antigen processing and presentation of peptide antigen via MHC class I |
| IL7R | 10.3* | -2.1 | regulation of DNA recombination, signal transduction |
| EDNRA | 19.3* | 1.2 | respiratory gaseous exchange, glucose transport |
| PRR5L | 13.5 | -1.1 | TORC2 signaling, negative regulation of protein phosphorylation |
| PLK2 | 5.4* | -2.6* | mitotic cell cycle checkpoint, Ras protein signal transduction |
| S1PR1 | 18.3* | 1.3 | blood vessel maturation, cardiac muscle tissue growth involved in heart morphogenesis |
| CHRM3 | 17.9* | 1.3 | G-protein coupled acetylcholine receptor signaling pathway, signal transduction |
| C3orf80 | 2.4* | -5.2* | NA |
| DYSF | 66.4* | 5.9* | plasma membrane repair, vesicle fusion |
| SLC9A9 | 21.1* | 1.9 | ion transport, transmembrane transport |
| CD180 | 9.7* | -1.1 | positive regulation of lipopolysaccharide-mediated signaling pathway, B cell proliferation involved in immune response |
| UST | 17.4* | 1.7 | carbohydrate metabolic process, protein sulfation |
| ANTXR1 | 3.3* | -3.1* | signal transduction, actin cytoskeleton reorganization |
| GALNT16 | 11.2* | 1.1 | protein glycosylation |
| C3AR1 | 11.6* | 1.2 | metabolic process, complement receptor mediated signaling pathway |
| HEY2 | 14.2* | 1.5 | vasculogenesis, muscular septum morphogenesis |
| SERPINI1 | 12.9* | 1.4* | central nervous system development, peripheral nervous system development |
| NA | 11.0* | 1.2 | NA |
| WIPF3 | 12.6* | 1.4 | Fc-gamma receptor signaling pathway involved in phagocytosis, innate immune response |
| PPFIA2 | 7.3* | -1.2 | signal transduction, cell-matrix adhesion |
| KCNMA1 | 19.2* | 2.2* | blood coagulation, synaptic transmission |
| MMP2 | 3.6* | -2.3* | extracellular matrix disassembly, extracellular matrix organization |
| TLR7 | 8.7* | 1.0 | toll-like receptor signaling pathway, toll-like receptor 9 signaling pathway |
| ZC3H12D | 2.8* | -3.0* | negative regulation of cell growth, negative regulation of G1 |
| CHODL | 14.3* | 1.8 | inflammatory response, antigen processing and presentation of peptide antigen via MHC class I |

Note: The values indicate the average fold changes in gene transcription of M0 and iDC compared to THP-1 monocytes based on 3 parallel cell-based experiments. *q < 0.05 indicates the significance calculated by intensity-based moderated t-statistics (IBMT). The table shows the 25 genes with the largest delta in fold changes between M0 and iDC with increased transcription in M0.
