# Supplementary material for: A THP-1 Cell Line-Based Exploration of Immune Responses Toward Heat-Treated BLG
Source: Front Nutr. 2021 Jan 13;7:612397. doi: 10.3389/fnut.2020.612397 (PMC7838438; doi:10.3389/fnut.2020.612397)
Supplement: Supplementary file 2 [file Table_2.docx]

**Table S2.** Top 25 genes that appear typical for iDC when compared to M0

| **Gene name** | **M0** | **iDC** | **Gene Ontology Description** |
| --- | --- | --- | --- |
| SERPINB4 | -3.3* | 515.0* | negative regulation of peptidase activity, regulation of proteolysis |
| SERPINB3 | -1.1 | 135.2* | response to virus, negative regulation of peptidase activity |
| AQP9 | 2.8* | 243.9* | metabolic process, transport |
| CCL22 | -1.5 | 51.5* | cell-cell signaling, response to virus |
| SERPINB12 | 1.3 | 70.7* | negative regulation of protein catabolic process, negative regulation of endopeptidase activity |
| SERPINB13 | -1.2 | 45.9* | response to UV, regulation of proteolysis |
| KCNK3 | 1.3 | 65.1* | potassium ion transport, synaptic transmission |
| EHF | 1.8 | 60.7* | multicellular organismal development, cell proliferation |
| SCIMP | -2.2 | 15.0* | positive regulation of ERK1 and ERK2 cascade |
| SLC1A2 | 1.0 | 32.2* | ion transport, synaptic transmission |
| INHBE | 1.2 | 35.8* | growth |
| CD274 | -1.4 | 20.0* | T cell costimulation, positive regulation of T cell proliferation |
| NR4A3 | 1.4 | 38.5* | biological_process, transcription initiation from RNA polymerase II promoter |
| NPR1 | 1.5 | 41.3* | positive regulation of renal sodium excretion, positive regulation of urine volume |
| ALDH1A2 | 7.8* | 211.9* | retinoic acid metabolic process, negative regulation of cell proliferation |
| SPINT2 | -2.3* | 10.7* | cellular component movement, negative regulation of endopeptidase activity |
| MMP7 | 3.5* | 78.4* | extracellular matrix disassembly, extracellular matrix organization |
| VCAM1 | 1.3 | 27.7* | cytokine-mediated signaling pathway, extracellular matrix organization |
| WISP1 | 1.0 | 20.9* | cell-cell signaling, signal transduction |
| TFPI2 | 12.1* | 242.6* | blood coagulation, negative regulation of endopeptidase activity |
| IL13RA2 | -1.1 | 18.3* | signal transduction, cytokine-mediated signaling pathway |
| GJB2 | -1.5 | 12.7* | transport, gap junction assembly |
| TGM2 | 9.3* | 156.1* | positive regulation of cell adhesion, apoptotic cell clearance |
| F3 | -2.4* | 6.7* | blood coagulation, blood coagulation, extrinsic pathway |
| ROR1-AS1 | 1.2 | 18.8* | NA |

Note: The values indicate the average fold changes in gene transcription of M0 and iDC compared to THP-1 monocyte based on 3 parallel cell-based experiments. *q < 0.05 indicates the significance calculated by intensity-based moderated t-statistics (IBMT). The table shows the 25 genes with the largest delta in fold changes between M0 and iDC with increased transcription in iDC.
