# Supplementary material for: A THP-1 Cell Line-Based Exploration of Immune Responses Toward Heat-Treated BLG
Source: Front Nutr. 2021 Jan 13;7:612397. doi: 10.3389/fnut.2020.612397 (PMC7838438; doi:10.3389/fnut.2020.612397)
Supplement: Supplementary file 3 [file Table_3.docx]

**Table S3.** Significantly activated or inhibited upstream regulators in M0 and iDC

| **Upstream Regulators** | **M0 (z-score)** | **iDC (z-score)** |
| --- | --- | --- |
| ECSIT | 3.47 | 3.74 |
| CD40LG | 2.77 | 3.25 |
| TNF | 2.68 | 3.29 |
| TLR4 | 2.38 | 3.13 |
| CD40 | 2.40 | 2.78 |
| IL17A | 2.42 | 2.61 |
| IL6 | 2.40 | 2.40 |
| TNFRSF1A | 2.20 | 2.42 |
| TREM1 | 1.63 | 2.12 |
| GAPDH | NA | 2.22 |
| TSLP | NA | 2.21 |
| mir-155 | -2.24 | -2.24 |
| mir-146 | -2.21 | -1.98 |
| TGFB1 | NA | -2.20 |
| LY6E | -1.96 | -2.18 |

Note: the z-score was calculated by IPA which represents the bias in gene regulation that predicts whether the upstream regulator exists in an activated or inactivated state. z-score > 2 (red) or < -2 (green) was considered to be significant. NA: data is not available.
