# Supplementary material for: A THP-1 Cell Line-Based Exploration of Immune Responses Toward Heat-Treated BLG
Source: Front Nutr. 2021 Jan 13;7:612397. doi: 10.3389/fnut.2020.612397 (PMC7838438; doi:10.3389/fnut.2020.612397)
Supplement: Supplementary file 4 [file Table_4.docx]

**Table S4.** Fold change of significantly differentially transcribed genes in H-glu-BLG and W-glu-BLG treated M0 when compared to non-treated M0

| **Sample** | **Gene name** | **FC** | **Gene Ontology Description** |
| --- | --- | --- | --- |
| H-glu-BLG | HLA-DQA2 | 2.0 | cytokine-mediated signaling pathway, antigen processing and presentation of exogenous peptide antigen via MHC class II |
|  | AMPD3 | 2.1 | purine nucleobase metabolic process, AMP catabolic process |
|  | SLC2A6 | 2.4 | transmembrane transport, glucose transport |
|  | SOD2 | 2.6 | release of cytochrome c from mitochondria, vasodilation by acetylcholine involved in regulation of systemic arterial blood pressure |
|  | NCF1C | 3.0 | oxidation-reduction process |
|  | CRIM1 | 3.3 | nervous system development, insulin-like growth factor receptor signaling pathway |
|  | EBI3 | 3.6 | humoral immune response, T-helper 1 type immune response |
|  | TNFAIP6 | 4.1 | cell-cell signaling, signal transduction |
|  | IL18R1 | 9.1 | signal transduction, immune response |
|  | ID1 | -3.1 | angiogenesis, transforming growth factor beta receptor signaling pathway |
|  | ID3 | -2.9 | multicellular organismal development, negative regulation of transcription, DNA-templated |
|  | MBNL2 | -2.1 | regulation of RNA splicing, regulation of alternative mRNA splicing, via spliceosome |
| W-glu-BLG | SUMO4 | 2.1 | protein sumoylation |
|  | TAF7 | 2.1 | spermine transport, negative regulation of transcription from RNA polymerase II promoter |
|  | ACKR3 | 2.1 | receptor internalization, chemokine-mediated signaling pathway |
|  | HLA-DQA2 | 2.2 | cytokine-mediated signaling pathway, antigen processing and presentation of exogenous peptide antigen via MHC class II |
|  | IDO1 | 2.3 | tryptophan catabolic process, female pregnancy |
|  | NCF1C | 2.5 | oxidation-reduction process |
|  | SOD2 | 2.6 | release of cytochrome c from mitochondria, vasodilation by acetylcholine involved in regulation of systemic arterial blood pressure |
|  | EBI3 | 3.2 | humoral immune response, T-helper 1 type immune response |
|  | TNFRSF9 | 3.2 | apoptotic process, negative regulation of cell proliferation |
|  | CRIM1 | 3.3 | nervous system development, insulin-like growth factor receptor signaling pathway |
|  | TNFAIP6 | 4.1 | cell-cell signaling, signal transduction |
|  | TNFSF18 | 4.5 | T cell proliferation involved in immune response, tumor necrosis factor-mediated signaling pathway |
|  | IL18R1 | 7.6 | signal transduction, immune response |
