# Supplementary material for: A THP-1 Cell Line-Based Exploration of Immune Responses Toward Heat-Treated BLG
Source: Front Nutr. 2021 Jan 13;7:612397. doi: 10.3389/fnut.2020.612397 (PMC7838438; doi:10.3389/fnut.2020.612397)
Supplement: Supplementary file 5 [file Table_5.docx]

**Table S5.** Fold change of significantly differentially transcribed genes in BLG, H-glu-BLG and W-glu-BLG treated iDC when compared to non-treated iDC

| **Sample** | **Gene name** | **FC** | **Gene Ontology Description** |
| --- | --- | --- | --- |
| BLG | TNFAIP6 | 3.7 | cell-cell signaling, signal transduction |
|  | TRBV5-5 | 2.4 | NA |
|  | SOD2 | 2.2 | release of cytochrome c from mitochondria, vasodilation by acetylcholine involved in regulation of systemic arterial blood pressure |
|  | LOC101929565 | -2.2 | NA |
| H-glu-BLG | OR8A1 | 3.2 | G-protein coupled receptor signaling pathway, axon guidance |
|  | CD6 | 2.3 | cell adhesion, receptor-mediated endocytosis |
|  | LINC01099 | -2.1 | NA |
|  | LOC101929565 | -2.1 | NA |
|  | MIR4797 | -2.2 | NA |
|  | AKTIP | -2.2 | positive regulation of protein phosphorylation, positive regulation of protein binding |
|  | UGT1A5 | -2.7 | metabolic process |
|  | MIR4451 | -3.3 | NA |
| W-glu-BLG | UGT1A5 | -3.2 | metabolic process |

NA: data is not available.
