# Supplementary material for: A THP-1 Cell Line-Based Exploration of Immune Responses Toward Heat-Treated BLG
Source: Front Nutr. 2021 Jan 13;7:612397. doi: 10.3389/fnut.2020.612397 (PMC7838438; doi:10.3389/fnut.2020.612397)
Supplement: Supplementary file 6 [file Table_6.docx]

**Table S6.** Significantly activated or inhibited upstream regulators in M0 incubated with BLG and L-glu-BLG (z-score > 2 or < -2)

| **Upstream Regulators** | **BLG (z-score)** | **L-glu-BLG (z-score)** |
| --- | --- | --- |
| ECSIT | 3.09 | 2.95 |
| IFNG | 3.09 | 2.75 |
| TLR4 | 3.09 | 2.58 |
| IL27 | 2.40 | 2.18 |
| TLR2 | 2.19 | 1.95 |
| TNF | 2.41 | NA |
| IL1B | 2.21 | NA |
| LY6E | -2.43 | -2.43 |

Note: z-score was calculated by IPA which represents the bias in gene regulation that predicts whether the upstream regulator exists in an activated or inactivated state. z-score > 2 (red) or < -2 (green) was considered to be significant. NA: data is not available.
