# Supplementary material for: A THP-1 Cell Line-Based Exploration of Immune Responses Toward Heat-Treated BLG
Source: Front Nutr. 2021 Jan 13;7:612397. doi: 10.3389/fnut.2020.612397 (PMC7838438; doi:10.3389/fnut.2020.612397)
Supplement: Supplementary file 7 [file Table_7.docx]

**Table S7.** Gene transcription of surface markers in M0 and iDC

| **Phenotypic markers** | **M0** | **iDC** |
| --- | --- | --- |
| CD1B | 1.1 | 4.6* |
| CD1C | 2.0 | 12.5* |
| CD1E | 1.9* | 2.1* |
| CD3G | 3.3* | -1.4 |
| CD4 | 1.2 | -3.6* |
| CD9 | 42.9* | 20.8* |
| CD11c | 15.7* | 21.2* |
| CD14 | 27.9* | 9.0* |
| CD22 | 87.9* | 36.1* |
| CD36 | 20.6* | 9.4* |
| CD38 | -12.3* | -7.4* |
| CD40 | 2.3* | 5.4* |
| CD44 | 2.7* | 6.4* |
| CD47 | 2.0* | 1.2 |
| CD52 | 27.9* | 41.7* |
| CD53 | 7.4* | 9.3* |
| CD55 | 3.4* | 2.7* |
| CD58 | 2.4* | 1.7* |
| CD59 | 4.8* | 4.7* |
| CD63 | 1.7* | 2.0* |
| CD70 | -4.6* | -8.5* |
| CD79A | -1.5 | 2.1* |
| CD81 | 2.5* | 2.0* |
| CD82 | 2.7* | 4.1* |
| CD83 | 7.4* | 16.1* |
| CD84 | 2.1* | 3.8* |
| CD86 | 1.8 | 9.5* |
| CD101 | 1.6 | -2.1 |
| CD109 | 10.1* | 2.2* |
| CD151 | 2.5* | 2.4* |
| CD163 | 17.9* | 6.6* |
| CD164 | 2.3* | 2.0* |
| CD180 | 9.7* | -1.1 |
| CD209 | 5.7* | 13.4* |
| CD226 | 2.3 | 16.6* |
| CD244 | -2.3* | -6.4* |
| CD274 | -1.4 | 20.0* |
| CD276 | 3.6* | 4.3* |
| CD300a | 5.6* | 3.9* |
| CD300lb | 2.8* | 26.0* |
| CD320 | -2.1* | -1.5 |
| HLA-A | 2.2* | 1.8* |
| HLA-B | 1.7* | 1.7* |
| HLA-C | 2.3* | 2.3* |
| HLA-DPA1 | -3.5* | -2.6* |
| HLA-DPB1 | -3.5* | -2.8* |
| HLA-DQB1 | -2.9* | -6.5* |
| HLA-DQB2 | -1.6* | -2.0* |
| HLA-DRA | -12.0* | -8.7* |
| HLA-DRB1 | 1.0 | -4.3* |
| HLA-DRB5 | -3.1* | -5.8* |
| HLA-DRB6 | 1.9* | 1.2 |

Note: The values indicate the surface markers’ gene transcription fold changes of THP-1 derived M0 and iDC compared to their control (exposed to culture medium) of N = 3 experiments, *p < 0.05. Only genes which has significant transcription level in at least one cell type were shown in the table.
