# Supplementary figures and images for: A THP-1 Cell Line-Based Exploration of Immune Responses Toward Heat-Treated BLG
Source: Front Nutr. 2021 Jan 13;7:612397. doi: 10.3389/fnut.2020.612397 (PMC7838438; doi:10.3389/fnut.2020.612397)

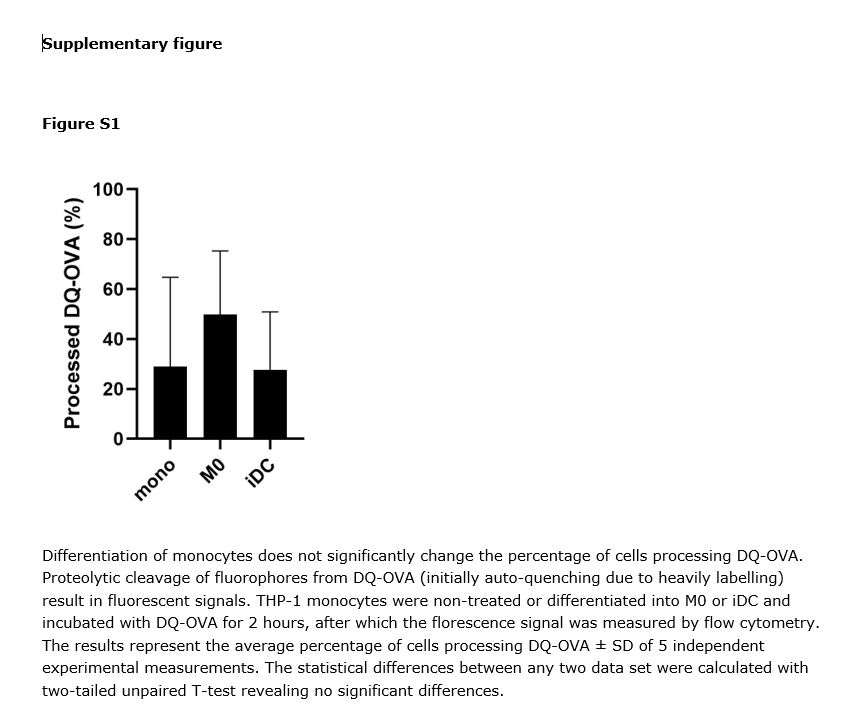

Supplement: Supplementary file 9 [file Image_1.jpg]
